# A Novel Sensor Based on a Single-Pixel Microwave Radiometer for Warm Object Counting: Concept Validation and IoT Perspectives

**DOI:** 10.3390/s17061388

**Published:** 2017-06-14

**Authors:** Federico Alimenti, Stefania Bonafoni, Luca Roselli

**Affiliations:** Department of Engineering, University of Perugia, via G. Duranti 93, 06125 Perugia, Italy; stefania.bonafoni@unipg.it (S.B.); luca.roselli@unipg.it (L.R.)

**Keywords:** microwave radiometer, antenna temperature, filling factor, warm target counting

## Abstract

Controlled measurements by a low-cost single-pixel microwave radiometer operating at 12.65 GHz were carried out to assess the detection and counting capability for targets warmer than the surroundings. The adopted reference test targets were pre-warmed water and oil; and a hand, both naked and wearing a glove. The results showed the reliability of microwave radiometry for counting operations under controlled conditions, and its effectiveness at detecting even warm targets masked by unheated dielectric layers. An electromagnetic model describing the scenario sensed by the radiometer antenna is proposed, and comparison with the experimental observations shows a good agreement. The measurements prove that reliable counting is enabled by an antenna temperature increment, for each target sample added, of around 1 K. Starting from this value, an analysis of the antenna filling factor was performed to provide an instrument useful for evaluating real applicability in many practical situations. This study also allows the direct people counting problem to be addressed, providing preliminary operational indications, reference numbers and experimental validation.

## 1. Introduction

The continuous expansion of the Internet of Things (IoT), allowing objects to be sensed and remotely integrated across wireless network infrastructures, covers a growing number of applications. The IoT encompasses several technologies able to gather information from the environment by employing both contact and remote sensors with low cost and low energy consumption features. With regard to remote sensors applied to the IoT scenario, an interesting application is the counting of warm targets and, in particular, of people.

The use of microwave (MW) radiometers for IoT applications aimed at detecting and counting targets warmer than their surroundings, especially when optically masked, is certainly a stimulating challenge. Several years ago, the idea of providing a method for the contactless grading and counting of agricultural products by using a MW radiometer at Ku band was proposed [1], but experimental results in the open literature are still missing. Currently, the counting issue is mainly focused on person counting, since many situations, such as security, safety and customer monitoring, can require people to be counted: specific non-restrictive examples being the estimation of queue length in shopping centres, the monitoring of entry points, train stations, bus terminals, and the evaluation of people flux in shops, stores, etc. Automated systems based on contact sensors have been developed for this over the years, such as pedestrian barriers and gateways. Several technologies that have the potential to count people, as described in [2], encompass video cameras, passive infrared (IR) cameras, infrared beam counters, piezoelectric pad, laser scanner. The counting performance of different technologies, including light-beam sensors, was evaluated in [3], whilst a laser sensor-based method was tested in [4]. Many commercial sensors are now available, but their accuracy under different environmental settings has to be assessed [4,5,6]. Non-contact counter systems usually employ visible and infrared sensors [7,8,9], but they exhibit some limitations, including, above all, the cost.

Even though significant progress has been made in sensor performance and machine learning detection algorithms, visible imaging is sensitive to variations in ambient lighting and scene colours. If a person is wearing the same shades of colours as the background, discrimination is difficult, and distinguishing a person from similar objects is generally not an easy task. Moreover, an automated counting visual camera only works accurately in the presence of suitable environmental light (natural or artificial); in case of darkness, shadows, fog and smoke, and variable lights in general, the system will likely fail.

Infrared imaging can overcome these limitations, but reliable measurements with IR systems primarily depend on the heating of the monitored external surface: the performance of human detection by thermal cameras is degraded in cases of low temperature difference between humans wearing a suit and the background, requiring suitable image processing procedures [10,11]. 

Overall, the above issues can be a crucial drawback when remote sensors are employed to detect and count targets warmer than their surroundings, especially when they are optically masked.

In this scenario, a system for counting warm targets using a low-cost non-imaging MW radiometer is proposed. Microwave radiometry is proven to be a reliable approach for the detection of hot spots masked by dielectric obstacles [12,13,14,15]. Microwave signals, in fact, can penetrate low-loss dielectric layers [13,15,16]; thus, microwave radiometers can detect a hot target even if it is optically masked, and also in cases where the masking layer is not heated yet. Moreover, MW systems may be effective, for instance, in situations where insufficient lighting, smoke and fog prevent the direct visualization of the scenario that are typical conditions, for example, in rescue scenarios or in outdoor night-time situation. From this perspective, a microwave radiometer with high sensitivity [17] can potentially detect the presence of people by exploiting the higher physical temperature of human beings with respect to their surroundings without being jammed by clothes [18].

The aim of this work is to assess the effectiveness and reliability of a MW radiometer for detecting and counting warm targets, resulting in considerable savings on hardware and software costs. For the first time, some experimental results on the applicability of a MW radiometer used as a low-cost non-contact and non-imaging counter sensor operating at 12.65 GHz are reported. This proof of concept, demonstrating the feasibility of an alternative technique to those already available, opens the way for a new branch of sensors for counting warm targets (including, but not limited to, people) and, to this extent, extends the availability of solutions for the problem of counting, eventually increasing the number of real situations that can be monitored by sensors independently connected to internet. Ultimately, it ends up making a valuable contribution towards the evolution of the so called “IoT ecosystem”, which is increasingly hungry for sensing technologies in more and more complex scenarios.

The paper is organized as follows: Section 2 describes the radiometer prototype; Section 3 illustrates the electromagnetic model, describing the operative scenario; Section 4 shows the radiometer observations for three controlled experiments (water, oil and hand), providing a comparison between measurements and model estimation. Finally, an analysis of the radiometer counting capability is reported, also addressing the people counting issue.

## 2. MW Radiometer Overview

Experimental results were carried out by means of a portable low-cost MW radiometer operating at 12.65 GHz, developed and realized at the Department of Engineering of the University of Perugia, aiming at performing field measurements of fire spots in different environmental conditions [12,13].

**Figure 1:**
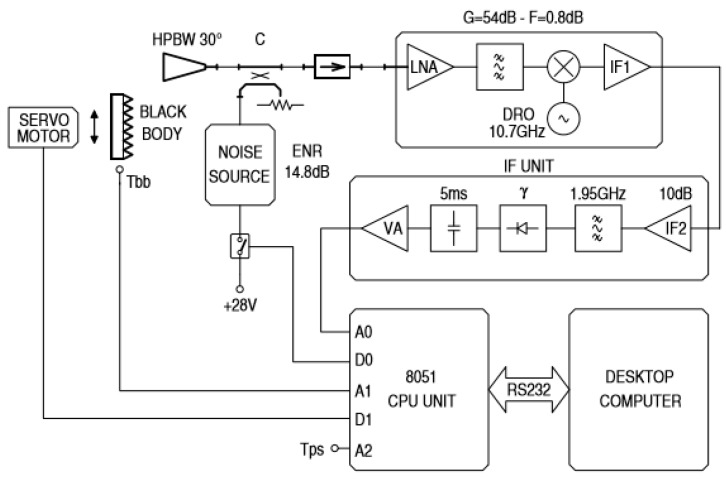
Block diagram of the microwave radiometer operating at the centre frequency of 12.65 GHz with a bandwidth of about 100 MHz. The receiver is based on a SAT-TV LNB. Figure from [13].

The radiometer scheme is shown in Figure 1: a detailed description of this sensor is provided in [12,13], and only the main features will be summarized in the following. It is a radiometer that can work both in total-power and noise-adding modes [19,20]. The receiver is built around a commercial low noise block (LNB) for satellite television applications, characterized by its high performance and low cost (less than € 20).

The noise-adding calibration circuitry (solid state noise source and WR75 directional coupler) allows for the correction of system gain fluctuations, while a motorized black body at ambient temperature (pyramidal microwave absorbing material in front of the radiometer antenna) is periodically used to correct the variations of the radiometric offset. Recently, solid-state noise sources have also been demonstrated in commercial CMOS technologies [21]. The realized prototype features a resolution of 70 mK with a 1 s integration time in total-power mode, and of 400 mK in noise-adding mode. The power consumption of the whole instrument is about 3 W.

**Figure 2:**
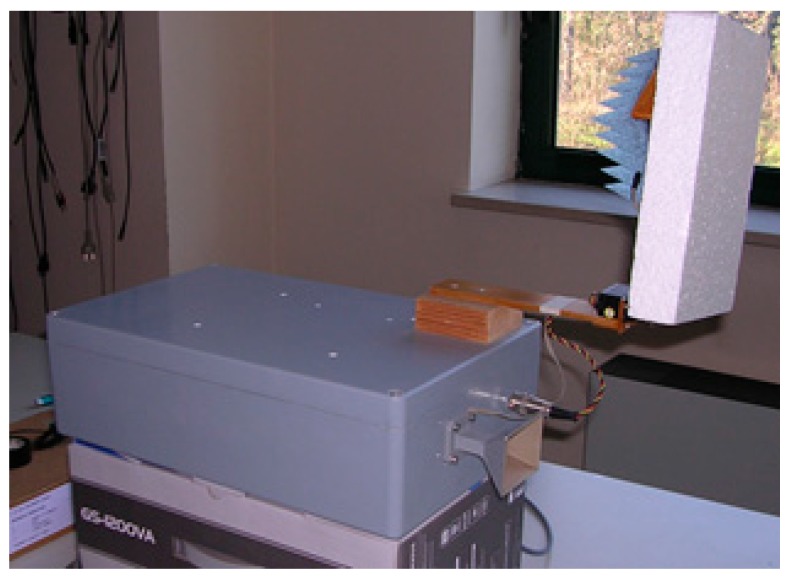
The microwave radiometer prototype

The tests described in the following sections were carried out using a standard gain horn antenna with a half-power beamwidth *β* = 30° and a gain of 15 dBi. Figure 2 shows a picture of the radiometer prototype.

This prototype is enclosed in a 39 cm × 24 cm × 12 cm box and has an overall mass of 2 kg, mostly due to the box itself.

**Figure 3:**
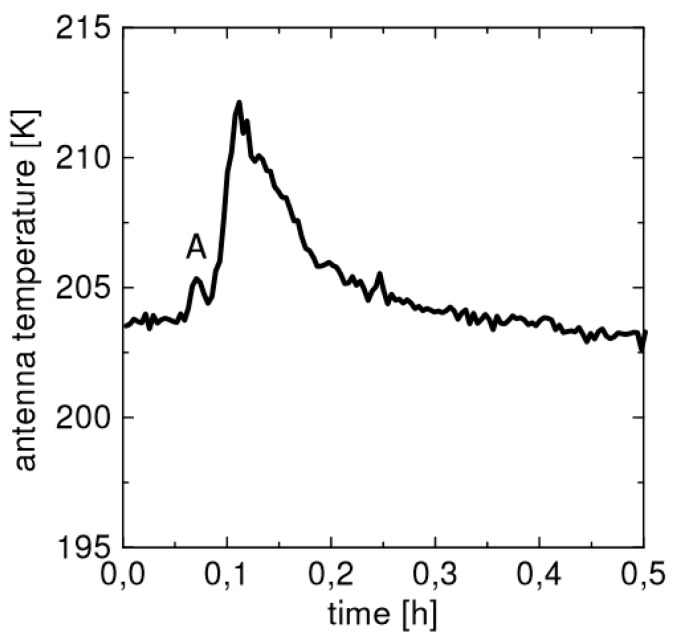
Antenna temperature recorded during an experiment carried out to detect a fire spot. The relative maximum *A* on the left of the main peak is caused by the person lighting the fire and falling inside the antenna beam, at a distance of 30 m from the antenna sensor. After [12].

The feasibility of millimeter-wave radiometry for human detection was demonstrated in [22] using a correlation radiometer consisting of two independent superheterodyne receivers with two separated antennas. The possibility of using a single microwave radiometer for people counting arose from the results described in [12] during an experiment aimed to detect fire spots with this radiometer prototype. As shown in Figure 3 [12], the relative maximum A was caused by a person inside the antenna beam when approaching to light the fire. Therefore, the radiometer detected the temperature increase due to the human body at a distance of 30 m from the antenna (a 60 cm offset dish antenna for SAT-TV application with *β* = 3°, in this case)

## 3. Scenario

**Figure 4:**
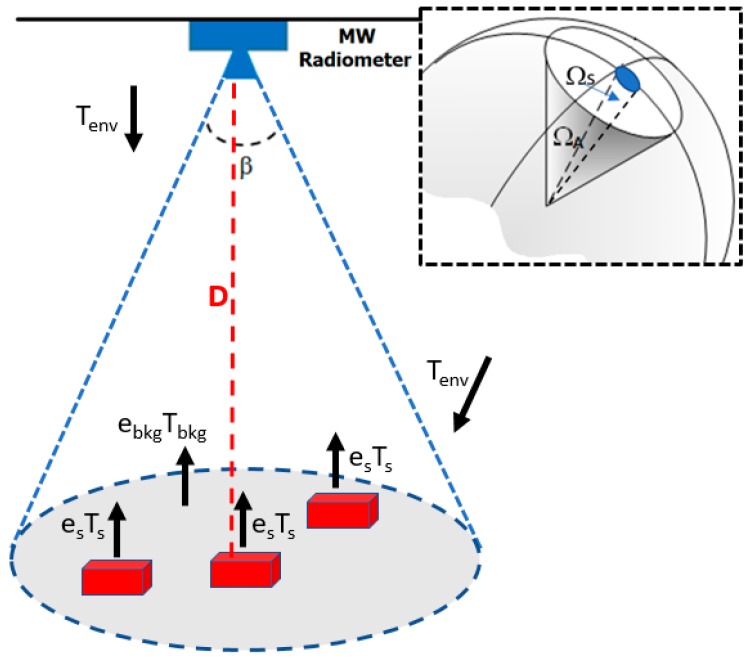
Scene sensed by a microwave radiometer; *β* is the antenna half-power beamwidth; D is the antenna-target distance; *e_s_T_s_* and *e_bkg_T_bkg_* are the brightness temperatures emitted by the target and background, respectively; *T_env_* is the contribution from the surroundings not observed by the antenna and going towards the antenna footprint (gray ellipse); the right inset depicts the filling factor *q*, i.e., the ratio of target solid angle (Ω_S_) to antenna solid angle (Ω_A_).

This section deals with the model describing the microwave radiative contributions sensed by a radiometer observing a scenario of interest for counting applications. We consider a single-pixel MW radiometer with the antenna half-power beam *β* covering the relevant target area as in Figure 4.

The measurement of a microwave radiometer is the antenna temperature *T_A_*, defined as the observed brightness temperature (*T_B_*(*θ, ϕ*)) distribution of the sensed scenario convolved with the antenna power pattern *F*(*θ*, *ϕ*) [23]:

(1) $$T_{A}=\frac{\iint_{4\pi }T_{AP}(\theta ,\varphi )F(\theta ,\varphi )d\Omega }{\iint_{4\pi }F(\theta ,\varphi )d\Omega }$$

where *θ* is the elevation angle, *ϕ* is the azimuth angle (rotation about beam centerline), *dΩ* = sin*θ dθ dϕ* is the elemental solid angle. The brightness temperature *T_B_* sensed by the microwave radiometer is the sum of two contributions:

(2) $$T_{B}=T_{Bdir}+T_{Bref}$$

where *T_Bdir_* represents the radiative contributions directly emitted by the sources inside the beamwidth, i.e., the background, the targets and the air, while *T_Bref_* comprises all the contributions (coming from the air and the environment not directly observed by the radiometer) reflected by the scenario and reaching the antenna beam.

Since the radiometer is placed at a distance of a few meters, the air emission and the related optical depth can be neglected in *T_Bdir_*, therefore the emissive contributions from the background and the targets are the main radiations characterizing *T_Bdir._* Each target emits a specific brightness temperature *T_BS_* as:

(3) $$T_{BS}=e_{s}T_{s}$$

where *e_s_* is its emissivity depending on dielectric properties of the material, surface roughness, frequency, polarization, and observation incidence angle [23]. *T_s_* is the physical temperature of each source.

In *T_Bref_* the reflectivity plays a key role: the energy conservation statement allows the surface reflectivity to be expressed as (1 − *e_s_*) [23] (p. 246). As well as in *T_Bdir_*, the air emission in *T_Bref_* can be neglected.

In order to explain and evaluate the microwave radiometer capability in detecting and counting targets warmer than the surroundings inside its antenna beam, the high sensitivity of a microwave radiometer has to be considered; in fact, it is able to detect a radiometric contrast in the *T_A_* even if the area occupied by a warm target is a small fraction of the whole antenna footprint. This aspect can be quantitatively explained by the so-called filling factor. The filling factor *q*, indeed, is defined as the power received from an extended source (i.e., the target) relative to that which would be received from a source having the same brightness but extending over the entire antenna power pattern [13,17,24].

Considering the aspects pointed out above, an operational model for the antenna temperature *T_A_* measured by a microwave radiometer can be derived for any sensor placement. The different radiative quantities contributing to *T_A_* can be expressed as the sum of two components: one related to the target of interest (warmer than the surroundings) and weighted by the filling factor *q*, and another related to the sensed scenario not occupied by the target and weighted by (1 *− q*):

(4) $$T_{A}={[e}_{s}{\;T}_{s}+(1-e_{s}{)T}_{env}\;]\;q+{[e}_{bkg}{\;T}_{bkg}+(1-e_{bkg}{)T}_{env}\;](1-q)$$

where *T_s_* and *e_s_* are the physical temperature and emissivity of the target; *T_env_* is the contribution coming from the surrounding environment (assumed as its physical temperature) not directly observed by the radiometer and going towards the surface sensed by the antenna radiometer; *T_bkg_* and *e_bkg_* are the physical temperature and emissivity of the background inside the antenna beam respectively (see also Figure 4).

Since the filling factor is actually proportional to the number of targets in the antenna beam, Equation (4) establishes a way to perform contactless counting by simply measuring *T_A_*.

Finally, the side-lobe contribution to the antenna temperature is an important factor, strictly related to the main beam efficiency ε_m_ of the radiometer antenna. Since the scene observed by the side-lobes can be modeled as an extended source [24], the antenna temperature increase due to the side-lobes can be obtained as the brightness temperature of the scene multiplied by (1 − ε_m_). A typical corrugated feed horn antenna is characterized by (1 − ε_m_) ~ 0.05. This means that an extreme physical temperature variation of ±20 K (scene observed by the side-lobes) with an emissivity equal to 0.5 will result in a side-lobe contribution of only ±0.5 K, i.e., close to the smallest variations considered in the present study, as found in the next sections.

## 4. Results

This section is divided in four sub-sections. Sub-section 4.1 describes the experimental set-up. Sub-section 4.2 shows the radiometer observations for three experiments. Sub-section 4.3 provides the comparison between measurements and model estimation. In Sub-section 4.4 an analysis of the radiometer target counting capability is addressed.

### 4.1. Experimental Set-Up

Three different experimental tests were carried out to assess the MW radiometer capability to detect warm targets under controlled conditions and to retrieve their number inside the antenna footprint. In the first two experiments, the targets were water and oil, warmed up and filling plastic containers, whilst in the third experiment a hand (in both naked and covered by a glove conditions) was observed. The radiometer operated in noise-adding mode (400 mK resolution) to ensure a rigorous real-time calibration.

**Figure 5:**
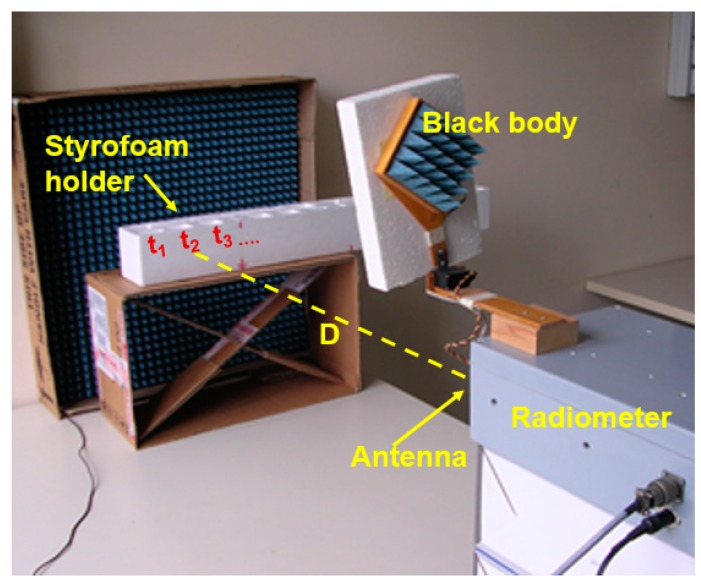
Experimental test setup. The radiometer antenna is placed at a distance *D* from the target; antenna-target position determines a normal incidence; a styrofoam sample holder was utilized to contain water and oil warmed up; t_1_, t_2_, t_3_, … are the sample holder bins sequentially filled up with water or oil, as described in Sub-section 4.2.

Figure 5 shows the experimental setup, where the background scenario is a pyramidal microwave absorbing material acting alike a black body radiator.

Water and oil were put into a sample holder made of styrofoam, as shown in Figure 5, with a target-antenna placement determining a normal incidence. The styrofoam had the advantage of thermally insulating the samples during handling and measurements, and enables a microwave transmissivity better than 0.99, as tested in [25]. Therefore, the styrofoam container can be considered “transparent” in the sensed scenario at microwaves (but opaque at infrared band).

As detailed in the next sub-section, the experiments were carried out with a scenario that was thermally controlled, with a constant distance antenna-target, and with targets of the same size.

During the tests, the settled integration time of the radiometer acquisitions was 1 s, a value not always adequate for capturing the dynamical behavior of moving objects. The radiometer adopted in the experiments, however, has a quite small observation bandwidth (only 0.8% of the center frequency). This means that, with a suitable receiver design, there is margin for a bandwidth increase of between 10 and 20 times. Consequently, assuming a constant integration time per bandwidth product, the integration time can be reduced to a value of between 50 to 100 ms without sacrificing the radiometric resolution.

### 4.2. Radiometer Measurements

With reference to Figure 5, the main parameters of the three experiments are reported in Table 1. The radiometer measurements were acquired during three different days, with *T_env_* and *T_bkg_* directly measured by temperature sensors.

**(1) Water Experiment**: the target is water warmed to 373.15 K, poured into the bins of the styrofoam sample holder (Figure 5). The cross-section dimensions of each bin are 7 cm (height) and 4 cm (width). Considering *D* = 0.79 m and *β* = 30°, the filling factor *q* for a single bin is 1.4%, approximating the horn antenna beam with its fundamental Gaussian mode [13,17,24].

**Figure 6:**
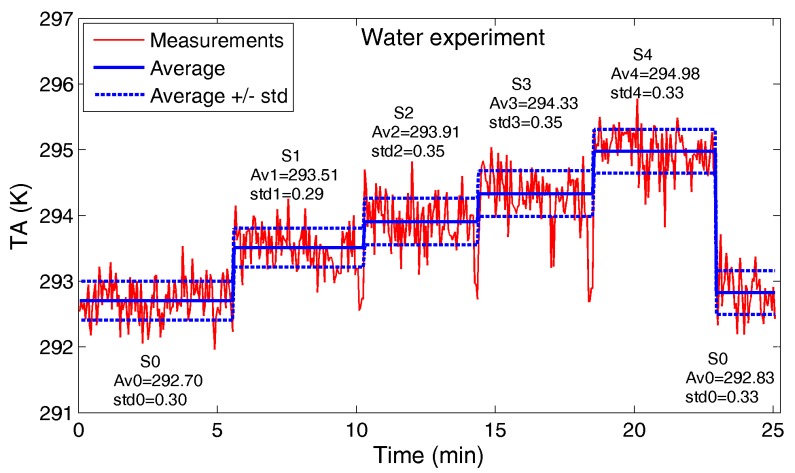
Water experiment: antenna temperature *T_A_* measured by the radiometer. For each setting (S0, S1, S2, S3 and S4), average (AvN) and standard deviation (stdN) are reported in Kelvin.

Figure 6 shows radiometer observations (*T_A_*) and related statistics in the following settings:

- S0: the target is absent;
- SN (N = 1, …, 4): N bins are filled up.

The peaks revealing a sharp decrease of *T_A_* in the transition between settings S1-S2, S2-S3 and S3-S4 are ascribed to the target handling phase (sample holder removal to fill up the subsequent bin). These transition phase measurements were not considered in the average and standard deviation computation reported in Figure 6.

The *T_A_* mean increment is around 0.8 K adding the first water bin (S1), and in the range 0.4–0.65 K adding each bin in sequence.

**Figure 7:**
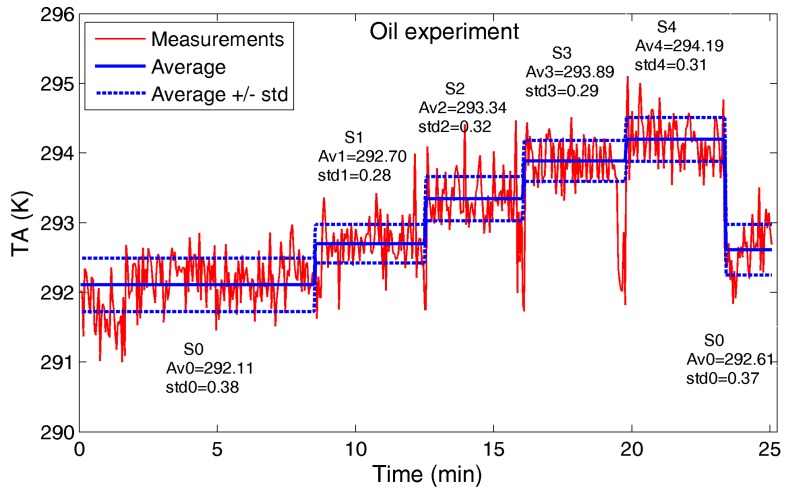
Oil experiment: antenna temperature *T_A_* measured by the radiometer. For each setting (S0, S1, S2, S3 and S4), average (AvN) and standard deviation (stdN) are reported in Kelvin.

**(2) Oil Experiment**: the target is vegetable cooking oil warmed to 341.15 K. The same settings S0 and SN (N = 1, …, 4) of the water experiment are proposed again, and the results are shown in Figure 7.

The *T_A_* mean increment is around 0.6 K adding the first water bin (S1), and in the range 0.3–0.65 K adding the bins in sequence.

For both experiments, even if the *T_A_* trend for the different settings highlights the incremental number of bins filled up by water or oil, the measurement standard deviation may create ambiguity in target counting. The small filling factor *q* of each bin (1.4%) provides a clear and detectable *T_A_* increment, even if an unambiguous counting capability would require a greater *q* (at the same target temperature): in Sub-section 4.4 an estimation of suitable filling factors will be provided.

**Figure 8:**
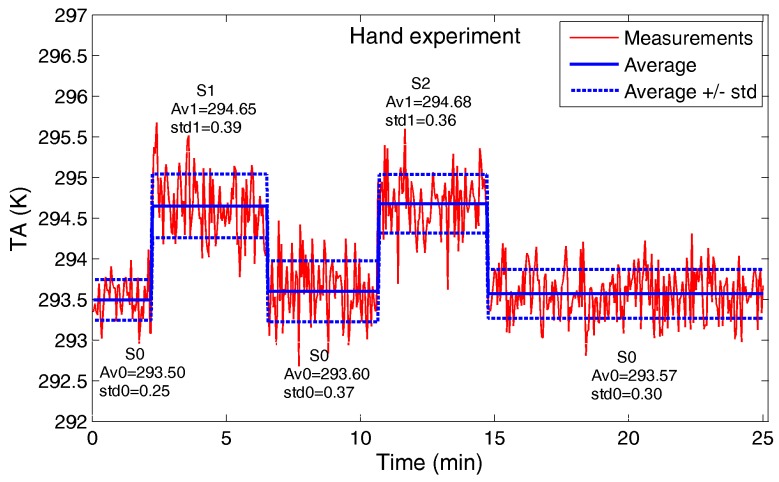
Hand experiment: antenna temperature *T_A_* measured by the radiometer. For each setting (S0, S1 and S2), average (AvN) and standard deviation (stdN) are reported in Kelvin.

**(3) Hand Experiment**: the target is a human hand, considered at a temperature of 309.15 K, in place of the styrofoam sample holder. Considering *D* = 0.79 m, *β* = 30° and the hand surface, the filling factor *q* is around 13%. Figure 8 shows *T_A_* and related statistics in the following settings:

- S0: the hand is absent;
- S1: the naked hand is present;
- S2: the hand wearing a synthetic fiber glove is present;

Since *T_A_* in S1 and S2 has the same behavior, this experiment confirms the microwave transparency of the gloves, and therefore the detection capability for a masked hand.

The presence of the hand produces a *T_A_* mean increment of around 1.1 K, greater than two times the standard deviation of the measurements.

### 4.3. Model and Measurement Comparison

Considering the model described by Equation (4) and the experiment parameters in Table 1, a simulation of the *T_A_* for the three experiments was performed. The model implementation requires an accurate choice of the target emissivity *e_s_*, mainly depending on frequency, incidence angle of observations and relative permittivity of the material. Table 2 reports the emissivity values to be considered in Equation (4) for normal incidence.

Water emissivity was computed as in [23] in consideration of the relative permittivity reported in [26] at the radiometer operative frequency and at a temperature of 373.15 K. Vegetable oil emissivity was computed following the typical microwave permittivity values found in [27]. The emissivity of a human body in the microwave band was considered to be 0.65, as reported in [22,28]. The microwave absorbing material ensures a background emissivity close to 1 (*e_bkg_* = 0.99 was assumed).

**Figure 9:**
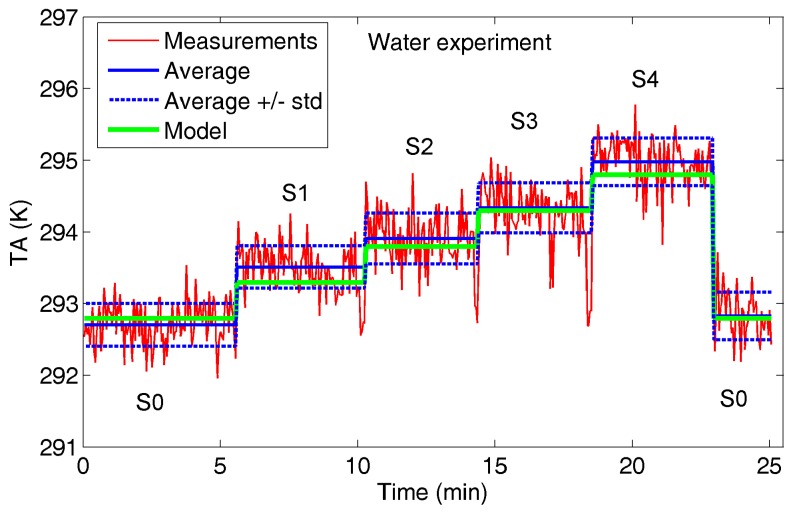
Water experiment: *T_A_* measured by radiometer (red and blue curves) and estimated by model (green curve).

**Figure 10:**
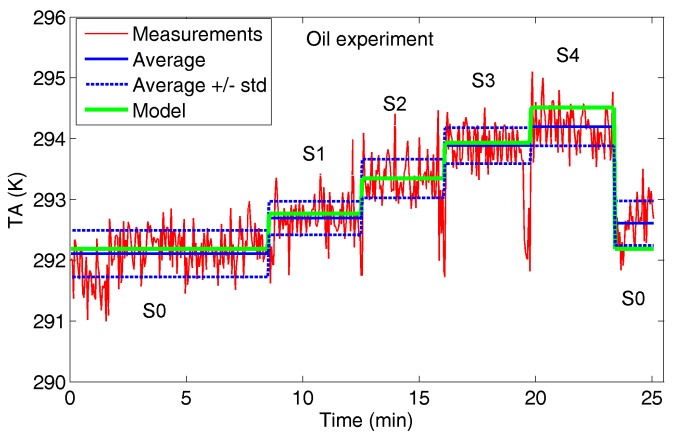
Oil experiment: *T_A_* measured by radiometer (red and blue curves) and estimated by model (green curve).

**Figure 11:**
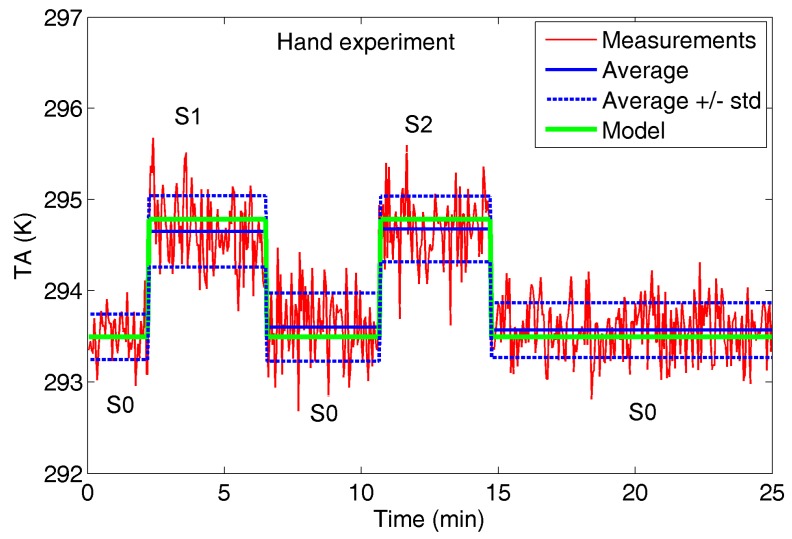
Hand experiment: *T_A_* measured by radiometer (red and blue curves) and estimated by model (green curve).

Figure 9, Figure 10 and Figure 11 show the comparison between radiometer measurements and model estimation for the three experiments.

The results prove the reliability of Equation (4) for describing the sensed scenario, and the soundness of emissivity computation from permittivity values in the literature (even if *e_s_* = 0.5 for water would provide a better fitting).

**Figure 12:**
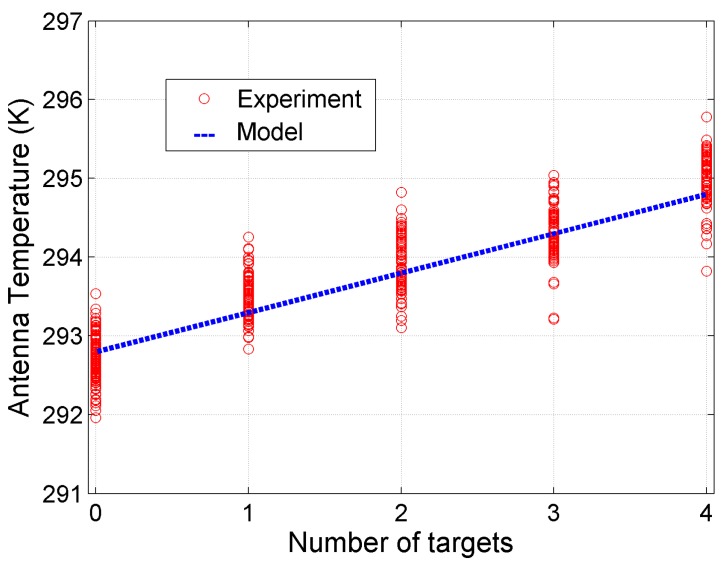
Water experiment: number of targets vs. *T_A_* measured by radiometer (red circles). In blue the *T_A_* estimated by model.

Overall, a way to perform a contactless counting by simply measuring *T_A_* with a single-pixel radiometer is shown with reference to the water experiment in Figure 12, in which the radiometer observations and the number of water samples are reported as a scatter plot with the model estimation superimposed.

### 4.4. Counting Capability

The standard deviations of the measurements reported in Figure 6, Figure 7 and Figure 8 suggest how reliable counting would benefit from an antenna temperature increment *ΔT_A_* of at least around 1 K, for each added sample. Applying the proposed model and, for the sake of simplicity, employing the same environmental conditions (*T_bkg_* and *T_env_*) reported in Table 1, an estimation of the filling factor *q* needed to ensure a *ΔT_A_* = 1 K at different target temperatures is proposed in Table 3. The two emissivities computed for normal incidence for water and oil (*e_s_* = 0.45 and *e_s_* = 0.85, respectively) were considered and assumed unchanged with temperature.

Applying Equation (4) to a human body, assuming *e_s_* = 0.65 and *T_s_* = 309.15 K, a filling factor of about 10% provides *ΔT_A_* = 1 K.

As expected, higher emissivities improve the detection capability, requiring a lower extension of the target with the same *D*, *β* and incidence angle *θ*.

It is important to underline the following points:

- The target emissivity grows for vertical (V) polarization when the incidence angle *θ* increases [23]; for instance, at *θ =* 60°, V polarization, water emissivity is around 0.65. For oil, at *θ =* 60°, V polarization, the emissivity is around 0.97.
- The water emissivity changes with temperature, and with reference to the relative permittivity reported in [26] and a temperature range of 303 K–373 K, the emissivity varies from about 0.4 to 0.45.

**Figure 13:**
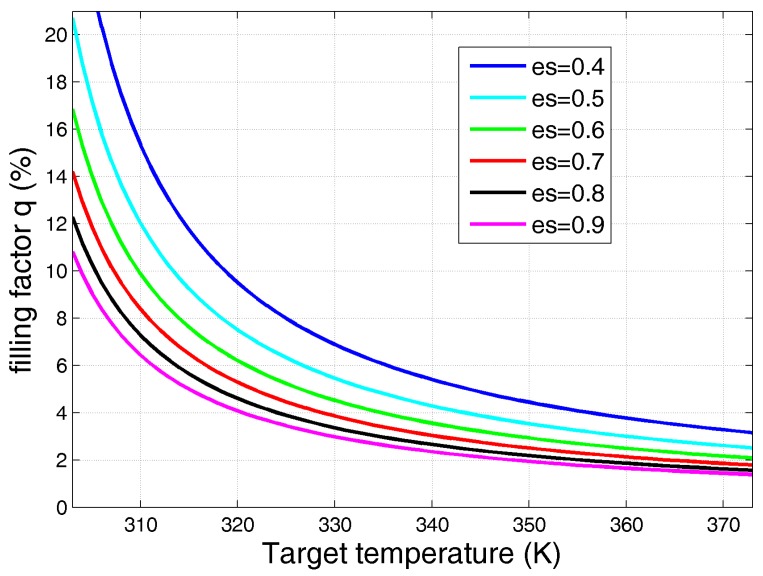
Filling factor *q* ensuring Δ*T_A_* = 1 K as a function of the target temperatures for different emissivity values (*T_env_* = 292.1; *T_bkg_* = 292.8; *e_bkg_* = 0.99).

An analysis useful for many practical situations in detecting/counting applications is the *q* computation, for Δ*T_A_* = 1 K, for different target temperatures and emissivity values, as shown in Figure 13.

A specific filling factor *q* can be achieved with different configurations (*D*, *β*, *θ* and geometrical dimensions of the target), following the computation in [13] (Appendix I). 

### 4.5. Counting People Perspectives

Since the counting of people is an interesting application in the IoT scenario, it is important to analyze the potential of MW radiometry for addressing this issue, exploring the possibility of extending sensing technologies solutions within the “IoT ecosystem”.

**Figure 14:**
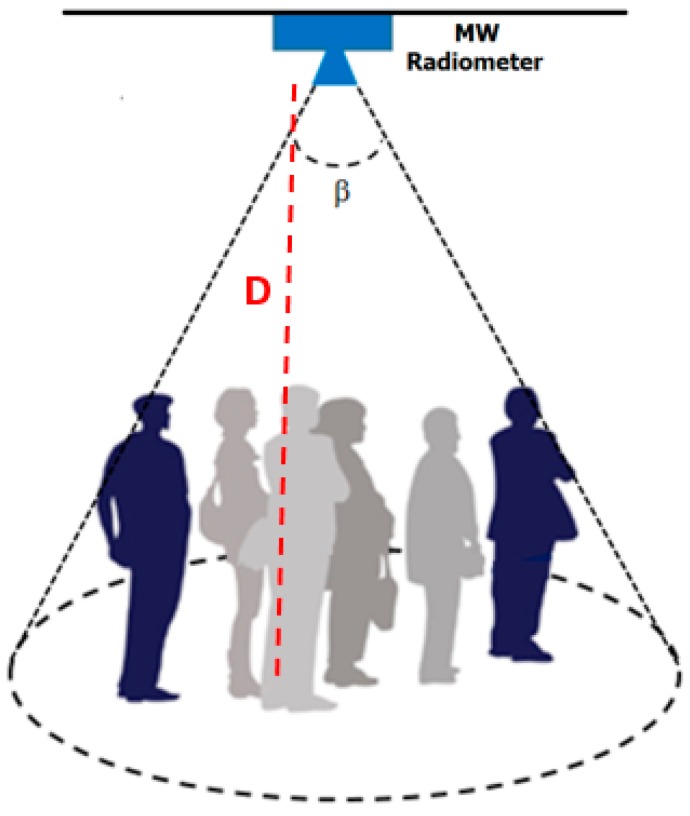
Scene sensed by a microwave radiometer mounted on the ceiling; *β* is the antenna half-power beamwidth. *D* is the distance antenna-floor.

**Figure 15:**
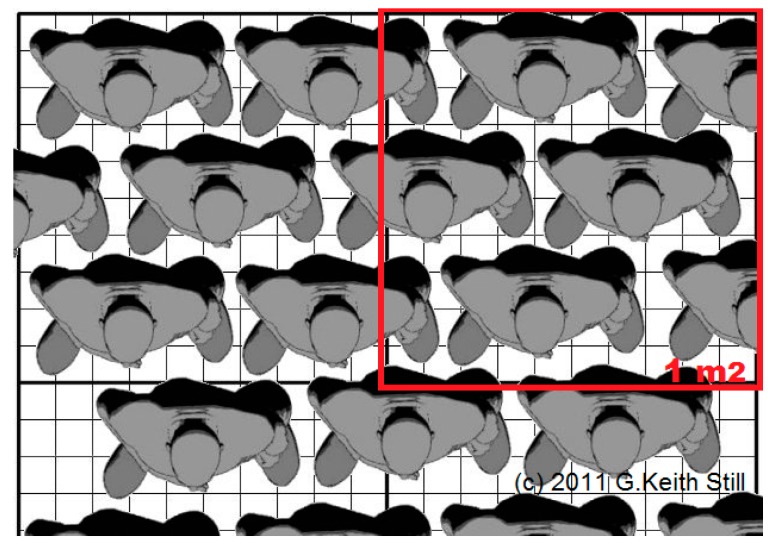
Picture from [30]: example of 5 standing people (adults) per square meter (red box), top view.

With reference to the scenario sensed by a microwave radiometer mounted on the ceiling, as depicted in Figure 14, different issues need to be addressed:

- Anthropometry of human beings [29]: differences in stature and related body proportions, mainly due to sex, ethnicity, and age, lead to a wide variety of situations inside the antenna beam. Moreover, the free-standing position or posture of a person is another variable;
- As described in [30], the upper limit of the number of people per square meter for a standing or moving crowd, considering safety limits and uncongested situations, can be fixed to 4/5 people (adults) per square meter (Figure 15);
- Location of a person inside the antenna beam: along the beam centerline, the main human body emission section is the shoulder/head area; towards the beam edge, the emission section varies but its evaluation also depends on the proximity of another person. At the edge, only a body fraction is observed.

An example of filling factor estimation for a single stationary human body positioned at the beam centerline of an antenna with *β* = 30° (vertically pointing, distance ceiling-floor *D* = 3 m), can be obtained exploiting static anthropometric data [29]. In Table 3, *q* is computed considering the 50th percentile of body measurements for British/US and Far-Eastern physical characteristics, both male and female, aged between 19 and 65 years. The antenna footprint area at the floor is around 2.03 m^2^, able to house about 10 persons.

The antenna configuration of Table 4 fulfils the counting requirements previously identified, i.e., approximately *q* = 10% and Δ*T_A_* = 1 K for a single person. It is important to point out the following operative features:

- *ΔT_A_* in Table 4 was estimated considering a background temperature *T_bkg_* = 293.5 K; assuming a floor temperature *T_bkg_* = 288.15 K, the filling factor providing *ΔT_A_* = 1 K decreases from *q* = 9.8% to *q* = 6.7%.
- The change of emissivity *e_bkg_* from 0.99 to 0.9, to simulate a floor, causes negligible variation in *q*.

Therefore, knowing the thermal conditions of the floor, an increment of *β* (or *D*) is possible, if necessary, to widen the sensed area.

**Figure 16:**
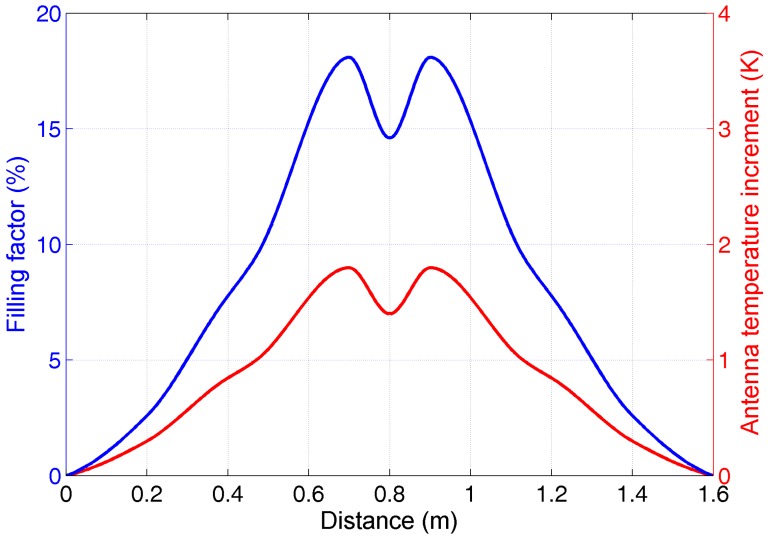
Filling factor *q* and antenna temperature increment for a single person (British/US male) crossing the antenna beam (*β* = 30°, vertically pointing, *D* = 3 m) and passing for the center (0.8 m). *T_env_* = 292.8; *T_bkg_* = 293.5; *e_bkg_* = 0.9.

In order to analyze the filling factor variation for a single person crossing the antenna beam, the estimations of *q* and of the correspondent *ΔT_A_* for the adults in Table 4 were performed: for the sake of conciseness, with a little loss of generality, the result obtained for a single adult British/US male passing through the beam center is reported in Figure 16. The antenna configuration (*β* = 30°, vertically pointing, *D* = 3 m) defines a circular footprint at the floor with a radius of 0.8 m.

The trend of *q* was estimated modelling a human body as a multi-parallelepiped (leg, trunk and head parallelepipeds using the 50th percentile of the anthropometric data in [29]) and computing the subtended solid angles of the body at different positions across the footprint diameter. The main human body emission is around the beam centerline, with the maximum nearly 10 cm away from the footprint center. In fact, at the center the main human body emission section is the shoulder/head area; moving few cm aside the footprint center, the subtended solid angle by the body includes not only a shoulder/head contribution, but also the projected contributions of the frontal section of the body. Towards the beam edge, *q* decreases, since only a body fraction is detected. 

**Figure 17:**
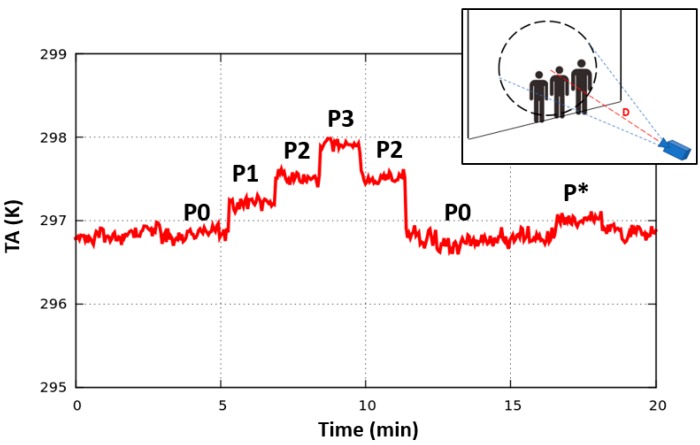
Antenna temperature *T_A_* measured by the radiometer for the stationary situation depicted in the right inset. Experiment settings: P0: persons are absent; P1: one person is inside the footprint; P2: two persons are inside; P3: three persons are inside; P*: one person is positioned at the edge of the antenna half-power beamwidth *β* (*β* = 30°, *D* = 4 m).

A first indoor test to analyze the potential people counting capability of a MW radiometer was carried out in a stationary situation (no moving people). In the experimental set-up, with respect to the scenario of Figure 14, the radiometric observation is horizontal instead of vertical, i.e., the radiometer points normally to the inner wall of the room, as depicted in the right inset of Figure 17. With the horn antenna having *β* = 30° and the distance antenna-wall *D* = 4 m, the antenna footprint area at the wall is around 3.7 m^2^. Three persons (2 adult females and 1 adult male) were positioned sequentially inside the footprint, standing stationary against the wall; the temporal evolution of the experiment is explained in Figure 17, in which the radiometer observations (*T_A_*) are shown in the following settings:

- P0: the persons are absent;
- PN (N = 1, …, 3): N persons are inside the antenna footprint, as depicted in the right inset.
- P*: only one person is present but positioned at the antenna beam edge.

The radiometer operated in total-power mode (70 mK resolution), and performed a post-processing gain calibration. The estimated filling factor *q* for each adult is between 8% (female) and 10% (male); these values are in the range of the ones of Table 4, making the choice of the horizontal set-up significant for this test, and also providing results interesting in the context of the vertical set-up discussed previously. In contrast to the experiments of Sub-section 4.2 and the previous simulations, *T_env_* and *T_bkg_* are around 297.5 K, indicating an environmental warming that reduces the thermal contrast with respect to a human body and, consequently, Δ*T_A_* (this test was carried out at the end of May, whilst the tests of Figure 6, Figure 7 and Figure 8 were performed in February 2017). Nevertheless, the test results show a clear antenna temperature increment associated with the progressive number of people inside the footprint (Δ*T_A_* around 0.4 K for each person).

## 5. Conclusions

The experimental approach has proven the effectiveness of microwave radiometry for counting issues under controlled conditions, and the potential use of a low-cost/single-pixel radiometer as a promising sensor for gathering information related to the detection and counting of targets warmer than their surroundings.

At the same time, the reliability of the electromagnetic model describing the scenario sensed by the radiometer antenna allowed for first guess estimation of the parameters required to design counting equipment. For instance, the filling factor, which ensures a suitable antenna temperature increment for each sample, or the role of the temperature and emissivity of the target. Both the model and the preliminary experimental results (hand test) also demonstrated the possibility of tackling the people counting instance. A great variety of actual situations, mainly related to the anthropometry of human beings and to the location of persons inside the antenna beam, may occur; in this work, we have analyzed some general cases and drawn typical operational indications, useful for future adjustments and tests. An experiment in an actual scenario reveals the MW radiometer as a promising technology in people counting issues, stimulating future investigations.

## Figures and Tables

**Table 1:** Experiment parameters. *T_s_* is the physical temperature of the target, *T_env_* is the contribution coming from the surrounding environment, *T_bkg_* is the physical temperature of the background inside the antenna beam, *D* is the distance antenna-target and *q* is the filling factor.

Experiment	*T_s_*	*T_bkg_*	*T_env_*	*D*	*q*
(1) Water	373.15 K	292.8 K	292.1 K	0.79 m	0.014
(2) Oil	341.15 K	292.2 K	291.5 K	0.79 m	0.014
(3) Hand	309.15 K	293.5 K	292.8 K	0.79 m	0.13

**Table 2:** Emissivity values.

*e_s_* (Water)	*e_s_* (Oil)	*e_s_* (Hand)	*e_bkg_*
0.45	0.85	0.65	0.99

**Table 3:** Filling factor estimation for Δ*T_A_* = 1 K at different target physical temperatures (*T_s_*) and with the same environmental conditions (*T_bkg_* and *T_env_*) of Table 1.

*T_s_* [K]	373 K	353 K	343 K	323 K	313 K	303 K
**q [%] (*e_s_* = 0.45)**	2.8	3.7	4.5	7.5	11.4	23.5
**q [%] (*e_s_* = 0.85)**	1.5	2.0	2.3	3.8	5.7	10.8

**Table 4:** Filling factor estimation and correspondent *ΔT_A_* for a single stationary British/US and Far-Eastern human body (male and female) positioned at the beam centerline. Anthropometric data: 50th percentile, age: 19–65 years [29]. Antenna parameters: *β* = 30°, vertical pointing, *D* = 3 m. *T_s_, T_env_* and *T_bkg_* as in Table 1, Experiment 3.

Anthropometric Data	Stature	Shoulder Breadth	Chest Depth	Shoulder Height	*q*	Δ*T_A_*
**British/US Male**	175 cm	47 cm	25 cm	143 cm	14.6%	1.4 K
**Far-Eastern Male**	167 cm	43 cm	20 cm	136 cm	9.8%	1.0 K
**British/US Female**	162 cm	40 cm	25 cm	132 cm	10.9%	1.1 K
**Far-Eastern Female**	154 cm	39 cm	21 cm	121 cm	7.9%	0.8 K
